# To Transfer or Not to Transfer an Electron: Anionic Metal Centers Reveal Dual Functionality for Polymerization Reactions

**DOI:** 10.3390/molecules30071570

**Published:** 2025-03-31

**Authors:** Andrei Evdokimov, Evangelos Miliordos

**Affiliations:** Department of Chemistry and Biochemistry, Auburn University, Auburn, AL 36849, USA; aze0056@auburn.edu

**Keywords:** metal anions, nucleophilic attack, electron transfer, radical polymerization, catalytic polymerization, solvent effects

## Abstract

Catalysts with anionic metal centers have recently been proposed to enhance the performance of various chemical processes. Here, we focus on the reactivity of ${\mathrm{Co}(\mathrm{CO})}_{4}^{-}$ for the polymerization of aziridine and carbon monoxide to form polypeptoids, motivated by earlier experimental studies. We used multi-reference and density functional theory methods to investigate possible reaction mechanisms and provide insights into the role of the negatively charged cobalt center. Two different reaction paths were identified. In the first path, ${\mathrm{Co}}^{-}$ acts as a nucleophile, donating an electron pair to the reaction substrate, while in the second path, it performs a single electron transfer to the substrate, initiating radical polymerization. The difference in the activation barriers for the two key steps is small and falls within the accuracy of our calculations. As suggested in the literature, solvent effects can play a primary role in determining the outcomes of such reactions. Future investigations will involve different metals or ligands and will investigate the effects of these two reaction paths on other chemical transformations.

## 1. Introduction

Transition metal complexes with electron-rich (anionic) metal centers have recently been shown computationally to hold promise for improved efficiency in various chemical transformations, such as the selective conversion of methane to methanol, capture and utilization of carbon dioxide, activation of molecular nitrogen, and recycling of perfluorochemicals [1,2,3]. For practical applications, it is important to identify ligands that can stabilize the negative charge on the metal center. One of the theoretically suggested ligands is carbon monoxide, which can act as a reservoir of electrons using its π-antibonding orbitals [3]. From the experimental side, ${\left[{\mathrm{Co}(\mathrm{CO})}_{4}\right]}^{-}$ has been identified as an intermediate species formed during the catalyzed copolymerization of carbon monoxide and aziridines to form poly-β-polypeptoids [4]. These authors demonstrated that the anionic metal center can induce the ring opening of aziridine via a two-electron nucleophilic attack. Their mechanism is supported by in situ measured infrared spectra and chemical kinetics analysis. Similar reactions occur with epoxides, imines, azetidines, and tetrahydrofuran [5,6,7,8]. Their proposed synthetic route is very different compared to the traditional routes of polypeptoids, which use amines and organic acids [9]. The importance of polypeptoids, which mimic the structure of peptides for biomedical applications and antimicrobial purposes, has been recently reviewed [10,11,12].

Inspired by the work of Darensbourg et al. [4], we carried out quantum chemical calculations to verify their proposed mechanism and provide insights into the molecular and electronic structures, as well as energetics. We were able to reproduce their suggested mechanism and also identify a different path leading to a living polymerization radical (LPR) mechanism, facilitated by single electron transfer (SET) [13]. As demonstrated later, unlike the typical SET-LPR processes, our proposed SET step is favored by non-polar solvents; thus, we will refer to it as the inverse solvent-response SET (ISR-SET). In the literature, solvent-responsive catalysts have been mentioned for various chemical processes [13,14].

In the following text, we first describe the computational methods employed, discuss our results, and finally provide our conclusions and repercussions of our findings.

## 2. Results and Discussion

### 2.1. Reaction Initiation Process

**Figure 1:**
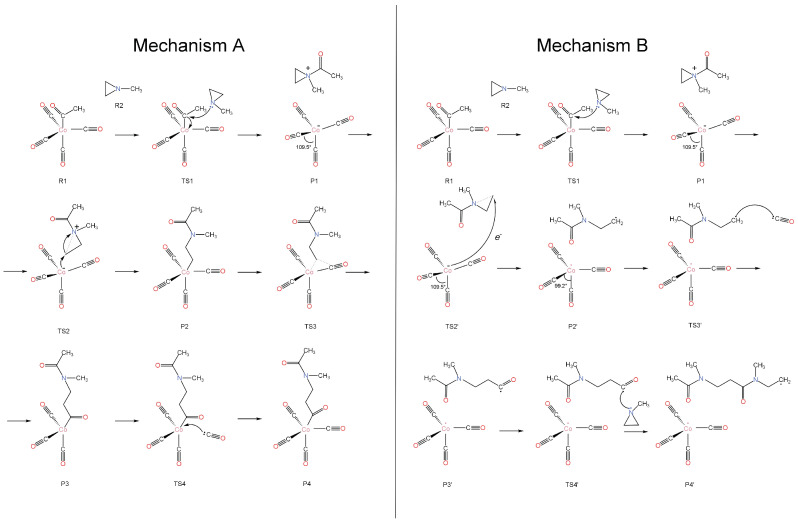
Schematic representation of mechanisms A and B.

The overall polymerization reaction under consideration is *n*CO + *n*(C_2_H_4_)NCH_3_ → {-C(O)CH_2_CH_2_N(CH_3_)-}*_n_*. The two carbon atoms in parentheses and the N in (C_2_H_4_)NCH_3_ (1-methylaziridine) form a three-membered ring. The two identified reaction mechanisms are shown schematically in Figure 1 and the corresponding energy diagrams are shown in Figures 3 and 4. The experimentally employed catalyst, (CO)_4_CoC(O)CH_3_, is used presently. In both mechanisms, the initial step involves the nucleophilic attack of aziridine on the electron-deficient carbon of the carbonyl group of the cobalt complex, leading to the generation of the [(C_2_H_4_)N(CH_3_)C(O)CH_3_]^+^ and [Co(CO)_4_]^−^ ion pair (P1 intermediate). This step goes through transition state TS1, which is 24.8 kcal/mol higher than the encounter complex of the reactants (R1⋯R2; see Figure 3).

The next step differentiates the two mechanisms. In mechanism A (suggested in Ref. [4]), a reverse nucleophilic attack occurs from the negatively charged Co center to one of the CH_2_ groups of aziridine, which triggers the cleavage of the ring of aziridine (see Figure 1). On the other hand, mechanism B involves an electron transfer from [Co(CO)_4_]^−^ to [(C_2_H_4_)N(CH_3_)C(O)CH_3_]^+^, which also leads to the cleavage of the ring and the production of two radical species, which in principle can initiate radical polymerization. In Figure 1, we show the polymerization reaction chain initiated by CH2CH2N(CH3)C(O)CH3•.

**Figure 2:**
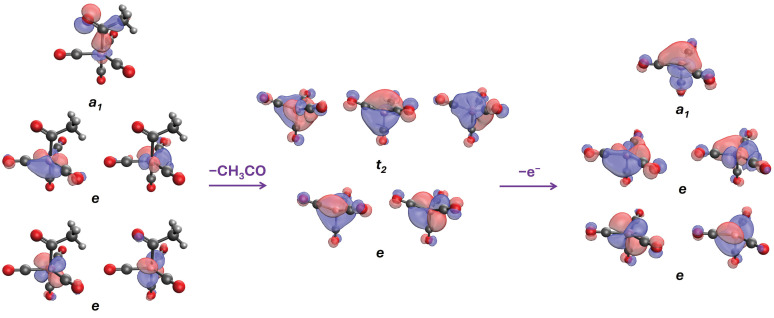
The highest energy orbitals of (CO)_4_CoC(O)CH_3_, (CO)_4_Co^−^, and (CO)4Co• (left to right).

The ground state of (CO)_4_CoC(O)CH_3_ is a closed-shell singlet adopting trigonal bipyramidal geometry. The metal center has nine valence electrons, which populate five *3d* orbitals (in energy order), as follows: ${3d}_{xy}^{2}$, ${3d}_{x^{2}-y^{2}}^{2}$, ${3d}_{xz}^{2}$, ${3d}_{yz}^{2}$, and ${3d}_{z^{2}}^{1}$. The first four orbitals form two doubly quasi-degenerate energy levels *e* (quasi-C_3v_), while the last one forms the $\sigma_{\mathrm{CoC}}$ bond ($a_{1}$) between the cobalt and the carbon atom of the carbonyl (see Figure 2). After the detachment of the acetyl group, ${\mathrm{Co}(\mathrm{CO})}_{4}^{-}$ forms a tetrahedral structure, and *3d* splits into the *e* and $t_{2}$ orbitals. The removal of an electron from ${\mathrm{Co}(\mathrm{CO})}_{4}^{-}$ leads to the trigonal pyramidal geometry, which now forms a perfect C_3v_ geometry with three equatorial, nearly co-planar carbonyl ligands. In all cases, there is at least a partial donation of electrons through π-back-bonding from the Co to the CO ligands. Figure 2 summarizes the changes in the structure and molecular orbitals during these processes.

### 2.2. Reaction Propagation: Mechanism A

**Figure 3:**
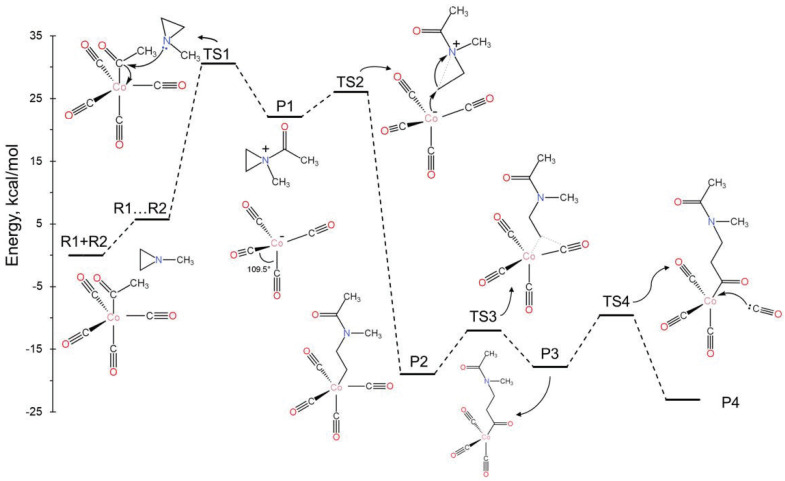
Free-energy landscape of the mechanistic pathway for the aziridine and carbonyl copolymerization reaction (mechanism A).

After the formation of P1 (see Figure 1), the electron-rich cobalt center can either approach the same carbon atom, thereby potentially returning to the initial reactants via TS1, or it can target one of the two carbons of the aziridine ring (TS2). The latter case leads to the highly stable intermediate P2, where the aziridine C-C bond is cleaved and a new Co-C is created. Then, one of the CO ligands shifts in between the Co and C of the newly formed bond (TS3; insertion reaction); finally, a new CO molecule enters the first coordination sphere of the cobalt (TS4). The complete free-energy diagram is shown in Figure 3. To make this diagram, the added CO molecule in the last step acted as an observer in all prior steps; thus, its energy was added to the energies of all previous species.

After the initial moderate-activation free-energy barrier (ΔG^‡^ = 24.8 kcal/mol or ΔE^‡^ = 17.0 kcal/mol), all steps are either spontaneous as they are highly exothermic or nearly thermoneutral with small activation barriers (<10.0 kcal/mol). The final product, P4, differs from R1 only in that the methyl group in R1 is replaced by a longer chain. Therefore, its reaction with an aziridine can initiate a new cycle. Since the rate-limiting step for every cycle corresponds to TS1, this mechanism supports the first-order dependence of the reaction rate on the concentrations of both aziridine and cobalt, as observed in the experiment [4].

### 2.3. Reaction Propagation: Mechanism B

**Figure 4:**
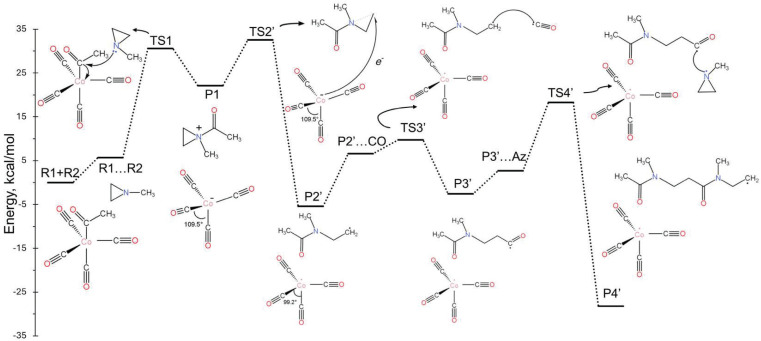
Free-energy landscape of the landscape of the mechanistic pathway for the aziridine and carbonyl copolymerization reaction (Mechanism B).

In this case, after P1 is formed, we observed that an electron transfer can occur from ${\mathrm{Co}(\mathrm{CO})}_{4}^{-}$ to the positively charged ammonium-type ion. The latter receives the electron and cleaves one of the N-C bonds of the aziridine ring. This is the same bond that cleaves during the nucleophilic attack of mechanism A, but now the open end of the created aziridine radical does not attach to the cobalt. In principle, the formed radicals, Co(CO)4• and CH_3_C(O)N(CH_3_)CH_2_CH2•, can re-combine to make P2 and switch back to mechanism A. Alternatively, they can launch radical polymerization reactions under different conditions. Specifically, comparing Figure 3 and Figure 4, we see that the electron transfer pathway has a higher $\Delta G^{‡}$ barrier for ${\mathrm{TS}}_{2}^{\prime }$ of 10.4 kcal/mol compared to 3.9 kcal/mol for TS2, while the P1/$P_{2}^{\prime }$ free-energy difference is smaller compared to P1/P2 (less exothermic), making the formation of $P_{2}^{\prime }$ less favorable in the gas phase. However, solvent effects (see Section 2.4) can favor mechanism B in three ways: (i) stabilize ${\mathrm{TS}}_{2}^{\prime }$, (ii) stabilize the produced radicals, or (iii) promote their rapid separation.

Figure 4 shows the energy diagram for radical polymerization initiated by the organic radical, CH_3_C(O)N(CH_3_)CH_2_CH2•. The addition of CO and CH_3_N(CH_3_CH_3_) to the organic chain is now more facile (smaller activation barriers). Specifically, the barrier for the incorporation of CO is 3.1 kcal/mol (P2’…CO/TS3’) vs. 6.9 kcal/mol for P2/TS3; for aziridine (Az), the barriers are 15.5 kcal/mol (P’…Az/TS4’) vs. 24.8 kcal/mol (R1…R2/TS1). The energies for P2’ and P2’…CO correspond to the energy difference between P2’ + CO (CO is an observer in the P2’ case) and P2’…CO (CO makes an encounter complex with P2’). Thus, their energy difference is due to a significant entropy change. In solution, this energy difference relates to the diffusion process rather than reaction energetics and can be incorporated into the diffusion rate constants. The same conclusion can be made for the P3’…Az/TS4’ case. The polymerization process can be terminated at any point by the combination of two radical chains or the intervention of Co(CO)4•. It should be emphasized that mechanism B goes through TS1 only once (at the initial step); thus, the rate law should be different from that of mechanism A.

**Figure 5:**
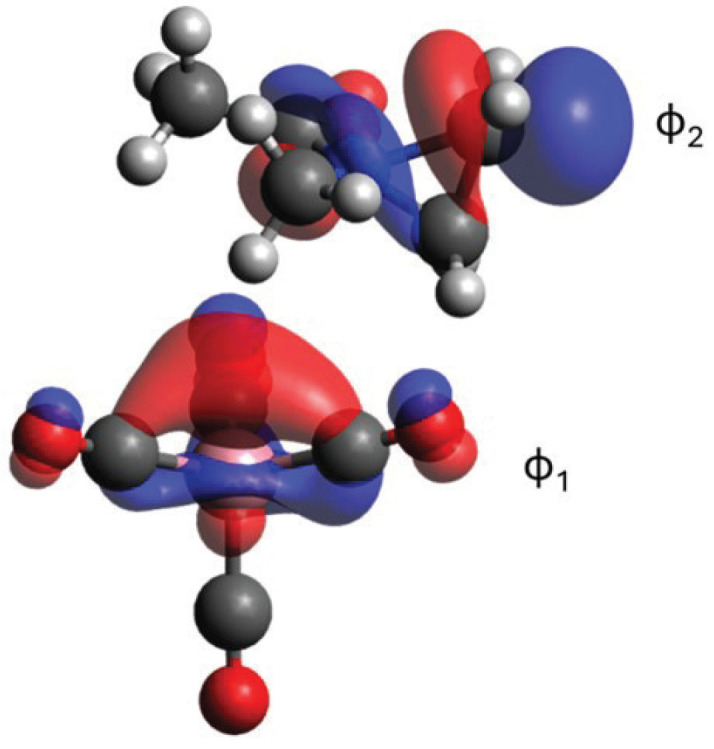
Select CASSCF orbitals of the TS2’ structure.

Among all reaction steps considered, the P1/TS2’/P2’ step is computationally the most challenging since it involves the transition from a closed shell singlet, ${\mathrm{Co}(\mathrm{CO})}_{4}^{-}$ CH_3_C(O)N(CH_3_)(C_2_H_3_)^+^, to an open-shell singlet system, CH_3_C(O)N(CH_3_)CH_2_CH2• and ${\mathrm{Co}(\mathrm{CO})}_{4}^{-}$. This transition requires the inclusion of three major electronic configurations in the wavefunction. The first one corresponds to $\phi_{1}^{2}\phi_{2}^{0}$ and the other two to $\phi_{1}^{1}\bar{\phi_{2}^{1}}$ and $\bar{\phi_{1}^{1}}\phi_{2}^{1}$, where $\phi_{1}$ is a localized orbital on Co and $\phi_{2}$ is the antibonding N-C orbital of aziridine (see Figure 5). Thus, the overall wavefunctions should be written as $c_{1}\phi_{1}^{2}+c_{2}\left(\phi_{1}^{1}\bar{\phi_{2}^{1}}-\bar{\phi_{1}^{1}}\phi_{2}^{1}\right)$, with the two coefficients changing along the reaction coordinates, that is, the coefficients ($c_{1}$, $c_{2}$) are (1, 0) and (0, $1/\sqrt{2}$) for P1 and P2’, respectively. DFT, as a single reference method, describes such electronic structure changes poorly, and we believe that it likely overestimates the activation barrier for TS2’.

**Figure 6:**
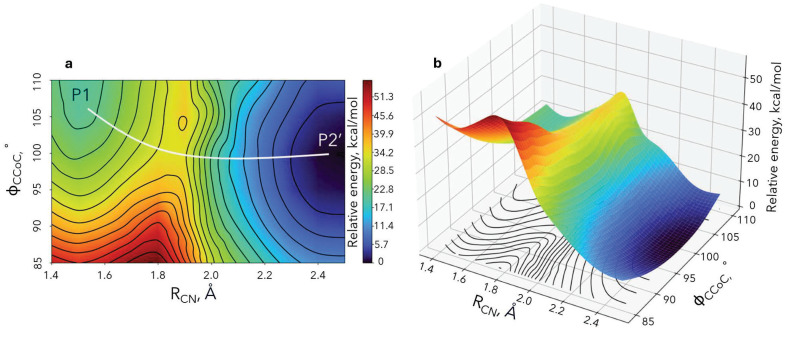
DFT/B3LYP potential energy surface for the electron-transfer step (bicubic interpolation is applied). The white line in the left plot shows the minimum energy path connecting the reactants (P1) and products (P2’).

To monitor both geometric and electronic structure changes along the reaction coordinate of the electron transfer step, we scanned over the two geometric parameters that primarily evolve. These are the N-C distance of the cleaved bond and the umbrella motion of Co(CO)_4_. The former N-C distance (R_CN_) increases from 1.49 Åto 2.47 Å (Figure 6a), while the C-Co-C angle ($\phi_{\mathrm{CCoC}}$) corresponds to the umbrella motion from 109.5^∘^ to 99.2^∘^ (Figure 6b). We calculated the minimum energy for each R_CN_/$\phi_{\mathrm{CCoC}}$ pair by optimizing over all other geometric parameters. The obtained two-dimensional potential energy surface and its contour mapping are shown in Figure 6.

**Figure 7:**
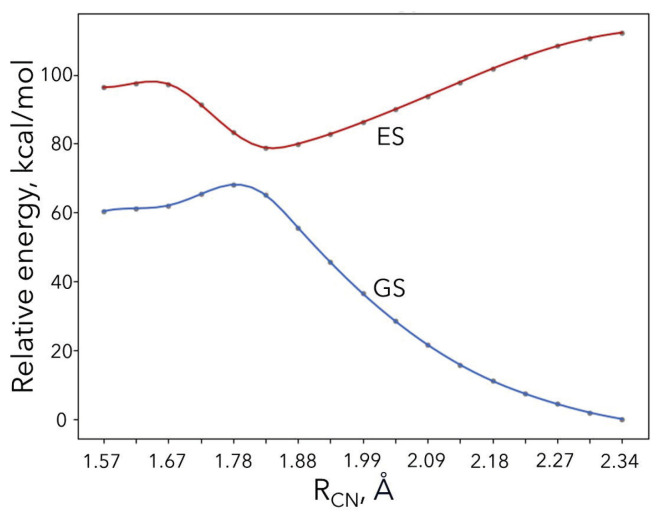
CASSCF electronic energy curves for the ground and first excited states along the electron transfer step of mechanism B.

Next, we carried out CASSCF calculations along the minimum energy path for the ground and first excited states (see Figure 7). The active space consisted of the $\phi_{1}$ and $\phi_{2}$ orbitals. The ground state for large R_CN_ values corresponds to the radical species (P2’), while the excited state corresponds to the corresponding ionic species. For shorter distances, the ground state character changes to the ionic species via the avoided crossing, right at the transition state geometry. The CASSCF electronic activation energy barrier is 7.7 kcal/mol, which is smaller than the DFT electronic energy barrier of 10.4 kcal/mol. The wavefunction at the transition state was found to indeed exhibit multi-reference character for the ground state ($c_{1}$ = 0.90, $c_{2}$ = 0.31) and excited state ($c_{1}$ = 0.44, $c_{2}$ = 0.63). More accurate post-CASSCF calculations (CASPT2) were not possible due to technical/convergence issues (possibly due to intruder states) in the transition state and products region. Overall, we believe that our CASSCF barrier is an upper limit since the optimal structure of the transition state may be different at a higher correlated level. Unfortunately, CASSCF geometries are known to be inaccurate, especially when a small active space is used, and CASPT2 calculations are not possible.

### 2.4. Mechanism A or B?

As already mentioned, the investigated polymerization reaction can follow two different pathways after forming the intermediate product, P1, and solvent effects play a decisive role. Solvent effects can affect the energy of the transition states (TS2 vs. TS2’) or the respective products, and can also facilitate the rapid separation of the two radical species formed in mechanism B. In the review by Rosen et al. [13], the authors discussed how single-electron transfer living radical polymerization (SET-LRP) reactions are conducted almost exclusively in polar solvent mixtures. The employment of polar solvents, such as DMSO, accelerates SET-LRP reaction rates by stabilizing the charge separation of the ionic products formed during the SET reaction step. In our case, we have an inverse process: SET proceeds from anion ${\mathrm{Co}(\mathrm{CO})}_{4}^{-}$ to cation [(C_2_H_4_N(CH_3_)C(O)CH_3_]^+^ with the formation of radical ${\mathrm{Co}(\mathrm{CO})}_{4}^{-}$ and CH_3_C(O)N(CH_3_)CH_2_CH2• species. This means that non-polar solvents stabilize the products and destabilize the reactants in this SET reaction. If a more polar solvent is used, then the ${\mathrm{Co}(\mathrm{CO})}_{4}^{-}$ and [(C_2_H_4_N(CH_3_)C(O)CH_3_]^+^ species would be stabilized, making the SET less likely to occur (and, thus, promoting mechanism A). This conclusion is clearly supported by the experimental work of Darensbourg et al. [4], where the rather polar 1,4-dioxane solvent was used and only mechanism A was observed. Therefore, mechanism B (radical polymerization) will proceed only with a suitable non-polar solvent.

Other important factors that could facilitate either mechanism A or B are the transition metal species used in the metal-anion catalyst complex and their ligand coordination. By varying metals, coordination, and ligands, we can tune the electron affinity of the ${\mathrm{ML}}_{n}^{-}$ complex, thus either stabilizing or destabilizing the anion–cation pair. This reactivity can be exploited for other chemical transformations beyond the copolymerization reaction described here.

## 3. Methods

We carried out both density functional theory (DFT) and wavefunction multi-reference calculations. The B3LYP hybrid functional [15] was combined with the aug-cc-pVTZ correlation consistent basis sets [16,17] to optimize the geometries for all reactants, intermediates, transition states, and products. Harmonic vibrational frequencies were calculated to identify the nature of all stationary structures. All reactants, products, and intermediates were found to have only real frequencies, while each transition state bore one imaginary frequency corresponding to a normal mode connecting the expected intermediates. The Complete Active Space Self-Consistent Field (CASSCF) method was used for the SET reaction step to properly describe the spin dynamics. Free energies were calculated using the harmonic approximation at a temperature of 298.15 K and pressure of 1 atm, as implemented in Gaussian 16 [18]. All DFT calculations were performed with Gaussian 16 software, and all CASSCF calculations were conducted using the Molpro program [19,20,21].

## 4. Summary and Conclusions

Inspired by the experimental work of Darensbourg et al. [4], we investigated the polymerization mechanism of carbon monoxide and aziridine facilitated by the (CO)_4_CoCH_3_ complex. Two mechanisms were identified. In mechanism A, the polymerization proceeds on the cobalt center, and in mechanism B, the polymerization follows a radical chain reaction, while the cobalt complex acts as the initiator. The key intermediate is the ion pair (CO)_4_Co^−^ + [(C_2_H_4_)N(CH_3_)C(O)CH_3_]^+^ (mechanism A), which can be converted to the radical pair (CO)_4_Co^•^ + ^•^(C_2_H_4_)N(CH_3_)C(O)CH_3_ (mechanism B) via the transfer of a single electron. The latter is expected to be favored in non-polar solvents, while the former is expected to be favored in polar solvents. This is the reverse effect (inverse SET-LRP) observed for similar polymerization reactions [13], where the electron transfer proceeds between neutral species.

Although we focused on a specific example in this work, our findings have more general implications for catalysts with negatively charged or electron-rich metal centers [3]. The use of polar solvents should be preferred to increase the stability of such catalysts. Given the small difference in the energy barriers of the two mechanisms (A and B), temperature and pressure can also be tuned to increase their stability. In the near future, we will investigate more metals and different ligands to monitor the relative preference for the two mechanisms. In addition, to gain a deeper and more realistic perspective on the dominance of mechanisms A or B, we will consider explicit solvent effects and conduct nuclear dynamics simulations.

## Data Availability

Data are contained within the article or Supplementary Materials.

## Supplementary Materials

The following supporting information can be downloaded at: https://www.mdpi.com/article/10.3390/molecules30071570/s1, Table S1. Optimized geometries (Cartesian coordinates in Å) and equilibrium electronic (E in a.u.) and free energies (G in a.u.) for all intermediate and transition states for the polymerization reaction.

## References

[1] Sader S., Miliordos E. (2022). Being Negative Can Be Positive: Metal Oxide Anions Promise More Selective Methane to Methanol Conversion. Phys. Chem. Chem. Phys..

[2] White M.V., Claveau E.E., Miliordos E., Vogiatzis K.D. (2024). Electronic Structure and Ligand Effects on the Activation and Cleavage of N_2_ on a Molybdenum Center. J. Phys. Chem..

[3] Androutsopoulos A., Sader S., Miliordos E. (2024). Potential of Molecular Catalysts with Electron-Rich Transition Metal Centers for Addressing Long-Standing Chemistry Enigmas. J. Phys. Chem..

[4] Darensbourg D.J., Phelps A.L., Gall N.L., Jia L. (2004). Mechanistic Studies of the Copolymerization Reaction of Aziridines and Carbon Monoxide to Produce Poly-*β*-peptoids. J. Am. Chem. Soc..

[5] Church T.L., Getzler Y.D.Y.L., Coates G.W. (2006). The Mechanism of Epoxide Carbonylation by [Lewis Acid]^+^[Co(CO)_4_]^-^ Catalysts. J. Am. Chem. Soc..

[6] Zhang Y., Ji J., Zhang X., Lin S., Pan Q., Jia L. (2014). Cobalt-Catalyzed Cyclization of Carbon Monoxide, Imine, and Epoxide. Org. Lett..

[7] Chai J., Liu G., Chaicharoen K., Wesdemiotis C., Jia L. (2008). Cobalt-Catalyzed Carbonylative Polymerization of Azetidines. Macromolecules.

[8] Liu G., Jia L. (2005). Cobalt-Catalyzed Carbonylative Copolymerization of N-Alkylazetidines and Tetrahydrofuran. Angew. Chem. Int. Ed..

[9] Qiu Z., Zhang M., Liu D., Shen X., Zhou W., Liu W., Lu J., Guo L. (2023). A Review on the Synthesis of Polypeptoids. Catalysts.

[10] Ganesh S.D., Saha N., Zandraa O., Zuckermann R.N., Sáha P. (2017). Peptoids and Polypeptoids: Biomimetic and Bioinspired Materials for Biomedical Applications. Polym. Bull..

[11] Nyembe P.L., Ntombela T., Makatini M.M. (2023). Review: Structure-Activity Relationship of Antimicrobial Peptoids. Pharmaceutics.

[12] Chongsiriwatana N.P., Patch J.A., Czyzewski A.M., Dohm M.T., Ivankin A., Gidalevitz D., Zuckermann R.N., Barron A.E. (2008). Peptoids That Mimic the Structure, Function, and Mechanism of Helical Antimicrobial Peptides. Proc. Natl. Acad. Sci. USA.

[13] Rosen B.M., Percec V. (2009). Single-Electron Transfer and Single-Electron Transfer Degenerative Chain Transfer Living Radical Polymerization. Chem. Rev..

[14] Zhou Y., Ryu E.H., Zhao Y., Woo L.K. (2006). Solvent-Responsive Metalloporphyrins: Binding and Catalysis. Organometallics.

[15] Becke A.D. (1993). Density-functional thermochemistry. III. The role of exact exchange. J. Chem. Phys..

[16] Dunning T.H. (1989). Gaussian Basis Sets for Use in Correlated Molecular Calculations. I. The Atoms Boron through Neon and Hydrogen. J. Chem. Phys..

[17] Kendall R.A., Dunning T.H., Harrison R.J. (1992). Electron Affinities of the First-Row Atoms Revisited. Systematic Basis Sets and Wave Functions. J. Chem. Phys..

[18] Frisch M.J., Trucks G.W., Schlegel H.B., Scuseria G.E., Robb M.A., Cheeseman J.R., Scalmani G., Barone V., Petersson G.A., Nakatsuji H. (2016). Gaussian˜16 Revision C.01.

[19] Werner H., Knowles P.J., Knizia G., Manby F.R., Schütz M. (2011). Molpro: A General-Purpose Quantum Chemistry Program Package. Wires Comput. Mol. Sci..

[20] Werner H.J., Knowles P.J., Manby F.R., Black J.A., Doll K., Heßelmann A., Kats D., Köhn A., Korona T., Kreplin D.A. (2020). The Molpro Quantum Chemistry Package. J. Chem. Phys..

[21] Werner H.J., Knowles P.J., Celani P., Györffy W., Hesselmann A., Kats D., Knizia G., Köhn A., Korona T., Kreplin D. MOLPRO, Version 2021.3, a Package of Ab Initio Programs, 2021. https://www.molpro.net.

