# Supplementary material for: To Transfer or Not to Transfer an Electron: Anionic Metal Centers Reveal Dual Functionality for Polymerization Reactions
Source: Molecules. 2025 Mar 31;30(7):1570. doi: 10.3390/molecules30071570 (PMC11990126; doi:10.3390/molecules30071570)
Supplement: Supplementary file 1 [file molecules-30-01570-s001.zip › molecules-3522789-supplementary.pdf]

Supporting Information for

To Transfer or Not to Transfer an electron: Anionic Metal Centers Reveal Dual Functionality for Polymerization Reactions

Andrei Evdokimov and Evangelos Miliordos*

Department of Chemistry and Biochemistry, Auburn University, Auburn, AL 36849-5312, USA

*emiliord@auburn.edu

Table S1. Optimized geometries (Cartesian coordinates in Å) and equilibrium electronic (E in a.u.) and free energies (G in a.u.) for all intermediate and transition states for the polymerization reaction.

Species							
R1				R2			
E = -1989.70568840		G = -1989.670018		E = -173.299678357		G = -173.228502	
Geometry				Geometry			
Co	-0.295821	0.012042	0.014593	N	0.155791	0.000029	-0.551888
O	0.261723	-0.240511	2.897754	C	-0.900042	0.743431	0.121144
O	2.225452	-1.302923	-0.339762	C	-0.900681	-0.743223	0.121106
O	-0.563983	-2.475161	-1.602976	H	-1.575630	1.292551	-0.521705
O	-0.076110	2.493668	-1.569992	H	-0.652251	1.238450	1.054970
O	-3.216221	0.361965	0.421663	C	1.457978	-0.000231	0.096611
C	2.627241	0.988894	0.119794	H	-1.576606	-1.291355	-0.522341
C	-0.460820	-1.530747	-0.983806	H	-0.653655	-1.238979	1.054653
C	-0.135622	1.539740	-0.948906	H	2.015315	0.883863	-0.214728
C	1.753363	-0.229533	-0.111348	H	2.015520	-0.883853	-0.215622
C	0.054616	-0.145343	1.780811	H	1.393236	-0.000747	1.194819
C	-2.100820	0.228095	0.257428				
H	2.614281	1.601197	-0.783899				
H	2.248372	1.600897	0.935498				
H	3.649888	0.669830	0.317060				
TS1				P1			
E = -2162.98147984		G = -2162.849819		E = -2162.99411417		G = -2162.863284	
Geometry				Geometry			
Co	-1.237757	-0.087995	-0.035513	Co	-1.664875	0.013316	0.057077
O	1.439040	1.518587	-1.153472	O	3.616613	-1.056936	1.036921
O	-3.940840	-1.203875	-0.303640	O	-4.464322	0.095232	0.962479
O	0.114516	-1.777813	-2.015003	O	-0.792793	2.650562	-0.850858
O	-1.799599	2.696281	-0.823419	O	0.070768	-0.676229	2.304329
O	-0.888848	-0.553854	2.839948	O	-1.490418	-1.927494	-2.133935
N	2.359896	-0.373903	0.164270	N	2.460511	0.415873	-0.337172
C	2.130011	-1.367204	1.219334	C	1.821184	0.526730	-1.673935
C	2.953300	-0.833952	-1.091914	C	2.070661	1.430648	0.713620
C	3.762826	-0.029857	-0.151270	C	3.399366	1.496524	0.091300
C	1.283453	1.797616	1.239854	C	1.917824	-2.067501	-0.342117
C	1.247351	1.060835	-0.070816	C	2.763831	-0.982567	0.214363
C	-2.889828	-0.768485	-0.197676	C	-3.374939	0.058511	0.606056
C	-0.387851	-1.098082	-1.234468	C	-1.195164	1.615753	-0.505072
C	-1.550948	1.621206	-0.518540	C	-0.655662	-0.425277	1.431093
C	-0.999677	-0.362083	1.710187	C	-1.580042	-1.168362	-1.266254
H	2.924577	-2.114125	1.211436	H	1.704914	1.579249	-1.912866
H	2.117769	-0.874454	2.188096	H	2.462607	0.046617	-2.408808
H	1.172646	-1.848294	1.046935	H	0.835018	0.071961	-1.630492
H	4.511375	-0.508712	0.463794	H	3.591501	2.222051	-0.684378
H	3.929094	1.012284	-0.383721	H	4.249636	1.128014	0.643995
H	2.553699	-0.339856	-1.965293	H	1.971157	1.002614	1.699531

H	3.113293	-1.898923	-1.169841	H	1.278207	2.089193	0.391316
H	2.267160	2.269527	1.335154	H	2.105526	-2.202542	-1.408063
H	0.529839	2.579670	1.204212	H	2.147939	-2.984600	0.190263
H	1.094721	1.161505	2.094742	H	0.860410	-1.811034	-0.227183
TS2				P2			
E = -2162.98534166		G = -2162.857066		E = -2163.06034361		G = -2162.928680	
Geometry				Geometry			
Co	-1.994369	0.005294	0.051319	Co	1.336272	-0.058410	0.039375
O	3.817050	-1.691023	-0.159189	O	-3.156985	-1.519350	-0.682267
O	-4.907143	0.197497	-0.307815	O	3.486346	0.719052	1.902742
O	-1.071329	-2.308291	-1.493900	O	1.510976	2.371358	-1.612141
O	-0.600864	2.364147	-0.963162	O	2.653130	-2.483401	-1.035529
O	-1.518651	-0.341782	2.926984	O	-0.562242	-0.596092	2.239712
N	2.728732	0.330522	-0.243857	N	-2.251298	0.521008	-0.333663
C	2.884362	1.800107	-0.322567	C	-2.283379	1.843043	0.272955
C	1.285433	-0.182978	0.320279	C	-1.391087	0.342097	-1.504514
C	1.642967	-0.280638	-1.083546	C	-0.189497	-0.580171	-1.325518
C	5.013554	0.153618	0.801068	C	-4.135791	-0.241272	1.082071
C	3.876218	-0.524449	0.097392	C	-3.152570	-0.471482	-0.049832
C	-3.772598	0.116161	-0.162763	C	2.657338	0.415010	1.187250
C	-1.455696	-1.409173	-0.877043	C	1.453470	1.442763	-0.952105
C	-1.228059	1.464339	-0.568623	C	2.165960	-1.549816	-0.605970
C	-1.721247	-0.206386	1.798957	C	0.131933	-0.366068	1.366317
H	1.912703	2.237091	-0.538826	H	-2.829579	2.561646	-0.347303
H	3.245943	2.188568	0.624594	H	-2.749044	1.809844	1.252090
H	3.578613	2.048432	-1.122995	H	-1.266804	2.211720	0.403320
H	1.908169	-1.250307	-1.476135	H	-1.087373	1.333079	-1.839146
H	1.206699	0.421847	-1.778191	H	-2.001855	-0.096486	-2.299094
H	0.701906	0.643694	0.684173	H	-4.761760	0.634237	0.905326
H	1.396785	-1.050368	0.952015	H	-4.766425	-1.121952	1.149957
H	5.472591	0.916235	0.171447	H	-3.622068	-0.099214	2.033737
H	4.669310	0.642088	1.713837	H	0.307067	-0.675648	-2.289451
H	5.752011	-0.601862	1.049202	H	-0.530488	-1.564368	-1.020995
TS3				P3			
E = -2163.04726264		G = -2162.917694		E = -2276.41996980		G = -2276.296803	
Geometry				Geometry			
Co	-1.599553	0.061478	-0.044156	Co	-1.869612	-0.639549	0.026939
O	2.680778	1.718948	0.191965	O	-1.353428	-2.097544	-2.456791
O	-3.542837	-0.063712	-2.266739	O	-1.552854	-1.976087	2.606186
O	-0.897193	-2.745323	-0.046421	O	-2.647827	4.348367	-0.249759
O	-1.171358	3.001391	0.044333	O	-4.832880	-0.571652	-0.102041
O	-2.844760	-0.149677	2.608129	O	-0.584517	1.284097	0.035010
N	2.881267	-0.528091	0.154184	O	3.776398	-0.027994	-1.754033
C	3.613435	-1.760661	-0.083354	N	3.627536	0.456764	0.442624
C	1.484578	-0.656030	0.580138	C	-1.549036	-1.458323	-1.530669
C	0.516655	-0.488429	-0.582533	C	-1.682077	-1.383640	1.636140
C	4.847479	0.885541	-0.372236	C	-3.248141	3.396278	-0.271738

<table>
<tr><td colspan="2">C 3.387388 0.734478 0.011871
C -2.766460 -0.001616 -1.435712
C -1.051046 -1.598940 -0.095153
C -1.324077 1.877546 0.006984
C -2.336700 -0.054495 1.592165
H 3.761742 -2.315190 0.847480
H 3.063758 -2.402807 -0.774758
H 4.584774 -1.557876 -0.519757
H 0.701433 -1.191646 -1.390467
H 0.592532 0.510174 -0.996173
H 1.288712 0.104548 1.331811
H 1.374834 -1.636051 1.044360
H 5.509771 0.369348 0.323178
H 5.039449 0.492677 -1.371858
H 5.077502 1.946170 -0.364072</td><td colspan="2">C -3.698548 -0.610740 -0.053628
C 4.148093 0.504484 1.798341
C 1.265487 -0.265939 0.172239
C 5.763956 -0.399255 -0.480668
C -0.168939 0.148403 0.085797
C 4.316869 0.020141 -0.656618
C 2.252417 0.905502 0.247292
H 3.507664 -0.062949 2.478248
H 1.356471 -0.931345 1.035049
H 5.851878 -1.273969 0.165392
H 5.142834 0.077018 1.846629
H 1.480356 -0.881095 -0.704806
H 4.197958 1.534558 2.161269
H 6.150112 -0.650744 -1.463260
H 6.371380 0.397580 -0.050637
H 1.991417 1.566128 1.072852
H 2.197069 1.480339 -0.673955</td></tr>
<tr><td colspan="2">TS4</td><td colspan="2">P4</td></tr>
<tr><td>E = -2276.41691366</td><td>G = -2276.286726</td><td>E = -2276.44469450</td><td>G = -2276.308113</td></tr>
<tr><td colspan="2">Geometry</td><td colspan="2">Geometry</td></tr>
<tr><td colspan="2">Co -1.889110 -0.376113 0.157869
O 3.755314 -0.699717 -1.623441
O -0.408292 1.636942 -0.524414
O -4.718169 -1.258263 0.346406
O -3.318219 3.026785 -1.390961
O -1.375516 -0.379791 3.032248
O -1.119310 -2.323711 -1.883648
N 3.636427 0.524567 0.267340
C 2.318828 1.016368 -0.129333
C 4.283262 -0.330598 -0.582051
C -0.184795 0.531629 -0.093661
C 5.673061 -0.808454 -0.203208
C 1.204440 0.012809 0.183773
C 4.144574 0.978806 1.549933
C -3.638771 -0.912721 0.269171
C -3.263454 1.992289 -0.952495
C -1.594901 -0.303521 1.912865
C -1.426111 -1.511060 -1.143679
H 2.329446 1.222345 -1.196563
H 2.137962 1.955781 0.391018
H 6.361243 0.021493 -0.040383
H 6.039496 -1.419473 -1.021922
H 4.303713 2.060571 1.545429
H 1.347110 -0.896815 -0.403767
H 5.085311 0.496337 1.788580
H 5.655567 -1.412717 0.705017
H 1.230460 -0.301090 1.231359</td><td colspan="2">Co -1.784989 -0.087365 -0.080544
O 3.371956 -1.652697 0.842278
O -1.936667 -1.295639 2.617279
O 0.272012 1.896515 -0.203859
O -0.155209 -1.205138 -2.269575
O -2.958540 2.632036 -0.301539
O -4.254427 -1.477867 -0.961842
N 3.143010 0.484917 0.153576
C 4.558115 -1.071948 -1.150096
C 3.650493 -0.778387 0.028930
C 3.526107 1.608383 -0.692974
C 2.361558 0.777304 1.356338
C 0.944821 0.198078 1.329206
C -0.778434 -0.769563 -1.420980
C -2.509191 1.592385 -0.221949
C -0.000479 0.865412 0.340692
C -1.837931 -0.826152 1.584586
C -3.313544 -0.946848 -0.612918
H 0.488466 0.311639 2.317642
H 2.875377 0.353749 2.219658
H 2.321942 1.858114 1.472122
H 5.439107 -0.429201 -1.153390
H 4.872011 -2.107891 -1.071005
H 4.039410 -0.930241 -2.099149
H 4.345874 2.184939 -0.251754
H 2.669033 2.264210 -0.825636
H 3.838835 1.262189 -1.672538</td></tr>
</table>

H	3.437399	0.745401	2.349996	H	0.981480	-0.872726	1.136761
TS2’				P2’			
E = -2162.97086551		G = -2162.846639		E = -2163.02308929		G = -2162.907171	
Geometry				Geometry			
Co	1.837080	-0.031696	-0.054067	Co	-1.787929	0.066120	-0.001713
C	3.289893	0.180709	-1.097998	C	-3.649347	0.198610	0.061634
O	4.204546	0.318215	-1.771749	O	-4.784275	0.277153	0.100707
C	1.415662	1.611182	0.500619	C	-1.371566	1.832928	-0.357327
O	1.071787	2.656189	0.850864	O	-1.117855	2.921049	-0.577438
C	0.519477	-0.729060	-1.026232	C	-1.473761	-0.535242	1.712704
O	-0.406080	-1.129800	-1.595228	O	-1.280734	-0.903606	2.774494
C	2.397687	-1.118050	1.271896	C	-1.622101	-1.125090	-1.398745
O	2.753773	-1.804203	2.120068	O	-1.521831	-1.858850	-2.265937
N	-2.521788	0.298505	0.348551	N	3.368928	-0.169277	-0.153884
C	-1.642163	0.089236	1.500525	C	4.594620	-0.952115	-0.107000
H	-2.099732	-0.591924	2.210215	H	4.456553	-1.869817	0.453818
H	-1.468427	1.043233	1.990470	H	5.370173	-0.369137	0.388769
H	-0.669323	-0.289381	1.159683	H	4.946348	-1.205074	-1.112311
C	-3.184886	-0.796225	-0.324249	C	2.117416	-0.690782	-0.019889
O	-3.829819	-0.562768	-1.312449	O	1.112813	0.011893	-0.121326
C	-3.053542	-2.150112	0.310863	C	1.996110	-2.174620	0.267181
H	-2.009011	-2.432734	0.431476	H	2.491228	-2.776758	-0.494663
H	-3.526757	-2.165471	1.294219	H	2.435603	-2.429197	1.232377
H	-3.551845	-2.865294	-0.335486	H	0.941033	-2.425896	0.288171
C	-3.559510	1.851672	0.345914	C	4.142835	2.058530	0.570552
C	-2.356288	1.545650	-0.426343	C	3.525670	1.245040	-0.508139
H	-3.486576	2.301853	1.322814	H	4.693467	2.955467	0.328398
H	-4.526007	1.595059	-0.055331	H	3.890502	1.869653	1.604123
H	-1.438953	2.060876	-0.172780	H	4.118610	1.313099	-1.426338
H	-2.483641	1.378982	-1.487347	H	2.523862	1.612703	-0.748736
P2’…CO				TS3’			
E = -2276.37036573		G = -2276.260890		E = -2276.37436831		G = -2276.255886	
Geometry				Geometry			
Co	1.955285	-0.045225	-0.048389	Co	2.055981	-0.035636	-0.060458
C	3.759216	0.421994	-0.190669	C	3.897935	-0.333729	-0.090227
O	4.858926	0.704130	-0.277148	O	5.021834	-0.513471	-0.106881
C	1.186413	1.621031	-0.273795	C	1.529539	-1.280871	-1.323417
O	0.709572	2.646973	-0.410919	O	1.213909	-2.044231	-2.107007
C	1.834704	-1.217997	-1.466956	C	1.717994	-0.442579	1.708294
O	1.755615	-1.941083	-2.345633	O	1.516353	-0.694708	2.801204
C	1.956738	-0.734771	1.663066	C	2.033929	1.731797	-0.575591
O	1.955447	-1.158368	2.721991	O	2.022346	2.825530	-0.899998
H	-2.627076	0.752267	0.150598	H	-2.286825	-0.413627	-1.690296
O	-0.872033	-0.623087	0.147511	O	-0.856114	0.325411	-0.100826
H	-4.190045	0.335969	0.833829	H	-3.768454	0.402133	-2.166638
C	-3.506036	0.117863	0.006552	C	-3.257949	-0.085702	-1.324017
H	-4.879430	1.204915	-1.377580	H	-4.288658	-2.014195	-1.600636

C	-1.707711	-1.521884	0.229586	C	-1.775920	1.069363	0.230678
C	-4.158933	0.404554	-1.296213	C	-4.060338	-1.258501	-0.861247
C	-2.463960	4.075164	0.408326	C	-2.676458	-2.514880	0.348463
N	-3.042239	-1.267992	0.130706	N	-3.028826	0.924448	-0.291365
H	-0.197624	-2.972687	0.555460	H	-0.504130	2.162779	1.523000
C	-1.276031	-2.960643	0.438265	C	-1.552125	2.174792	1.243182
H	-4.469608	-2.300628	1.307291	H	-4.275917	2.551187	-0.811434
H	-1.736278	-3.398544	1.324151	H	-1.800525	3.154932	0.835677
C	-4.080869	-2.275199	0.284056	C	-4.142511	1.816901	-0.010652
O	-1.997026	5.099248	0.384955	O	-2.874728	-2.535784	1.470586
H	-3.778905	-0.043656	-2.203222	H	-4.802593	-1.120625	-0.085587
H	-4.905569	-2.037711	-0.387245	H	-5.063666	1.240723	0.075163
H	-3.715856	-3.263710	0.026707	H	-3.997222	2.345557	0.924429
H	-1.541818	-3.582384	-0.417802	H	-2.155079	2.020662	2.138816
P3’				P3’…Az			
E = -2276.40072652		G = -2276.275497		E = -2449.70548055		G = -2449.495602	
Geometry				Geometry			
Co	2.177400	-0.123841	-0.021194	Co	-2.691753	-0.215916	0.238774
C	3.995921	-0.542019	-0.008518	C	-4.409321	-0.815473	0.658403
O	5.105552	-0.795352	-0.000155	O	-5.457128	-1.179958	0.911491
C	1.568736	-1.446502	-1.160977	C	-1.791583	-0.807006	1.742654
O	1.194624	-2.258097	-1.867011	O	-1.245383	-1.171591	2.672591
C	1.809070	-0.335147	1.775395	C	-2.423405	-1.155858	-1.325728
O	1.588149	-0.466811	2.885491	O	-2.260517	-1.738279	-2.292024
C	2.268126	1.586541	-0.699432	C	-3.028252	1.590634	0.127041
O	2.325657	2.643758	-1.123589	O	-3.235078	2.710493	0.060625
H	-2.150746	-0.571859	-1.526541	C	3.448204	0.976520	0.756654
O	-0.715030	0.415398	-0.154062	H	4.249122	1.429867	0.163941
H	-3.537875	0.272934	-2.223674	H	3.189592	0.015768	0.291302
C	-3.123355	-0.145828	-1.302646	C	4.015069	0.621205	2.110584
H	-4.147978	-2.010164	-1.564551	N	2.913337	-2.179625	-0.552225
C	-1.649868	1.150650	0.145322	C	3.289723	-2.569214	-1.905694
C	-4.067802	-1.238556	-0.794273	H	3.802831	-1.817328	-2.491688
C	-3.544036	-1.941684	0.443469	H	2.591314	-3.184725	-2.462745
N	-2.912058	0.935942	-0.343790	C	3.954599	-3.184606	-0.727701
H	-0.398299	2.323045	1.383691	H	4.955025	-2.885068	-0.443932
C	-1.440369	2.323443	1.082197	H	3.701475	-4.213675	-0.495384
H	-4.210893	2.444132	-1.049326	C	1.622102	-2.662287	-0.076025
H	-1.666771	3.272821	0.596008	H	1.363179	-3.650918	-0.479570
C	-4.027602	1.849207	-0.148971	H	0.848328	-1.950044	-0.359197
O	-4.137272	-2.250297	1.413498	H	1.644508	-2.729828	1.011939
H	-5.076731	-0.867541	-0.590387	O	5.112769	0.290042	2.387000
H	-4.936394	1.292774	0.080277	C	2.215285	1.878380	0.857929
H	-3.841202	2.523240	0.678207	N	1.690057	2.258246	-0.452485
H	-2.064862	2.243311	1.972290	C	2.326429	3.401297	-1.089433
				H	3.410613	3.298187	-1.038347
				H	2.052117	3.472727	-2.135459

				H	2.050509	4.335293	-0.590982
				C	0.596868	1.611706	-0.946132
				C	0.036146	2.074007	-2.277083
				H	0.757450	1.933038	-3.082947
				H	-0.844787	1.478503	-2.491816
				H	-0.242969	3.127580	-2.257347
				O	0.075957	0.677589	-0.337972
				H	2.459608	2.794270	1.400010
				H	1.429502	1.363530	1.401793
TS4’				P4’			
E = -2449.69062924		G = -2449.470845		E = -2449.76139593		G = -2449.544933	
Geometry				Geometry			
Co	2.821790	-0.606459	-0.034340	Co	2.748476	-0.750624	-0.056109
C	4.259238	-1.801123	-0.062544	C	4.004735	-2.132067	-0.088893
O	5.135246	-2.527940	-0.079904	O	4.770986	-2.973459	-0.108824
C	1.442588	-1.790576	-0.338864	C	1.197898	-1.749510	-0.034232
O	0.584600	-2.522527	-0.512013	O	0.235163	-2.360152	-0.007202
C	3.004171	0.026511	1.685868	C	3.241358	0.060126	1.521073
O	3.114456	0.404759	2.756449	O	3.544687	0.554815	2.503371
C	3.318930	0.488773	-1.439305	C	3.185518	0.093070	-1.643753
O	3.623062	1.159382	-2.308566	O	3.455188	0.609443	-2.622205
C	-2.703522	0.620926	0.197396	C	-2.503502	0.783876	0.247571
H	-3.138775	1.187913	1.036297	H	-2.955849	1.292363	1.104919
H	-1.933023	-0.027386	0.608807	H	-1.691383	0.172104	0.636437
C	-3.817055	-0.134390	-0.494722	C	-3.567526	-0.082760	-0.418898
N	-4.059930	-1.506792	0.067729	N	-3.969276	-1.201864	0.252334
C	-5.152099	-1.736772	1.278252	C	-6.204888	-2.242870	0.319449
H	-5.500153	-0.789830	1.657655	H	-6.653917	-1.375453	0.781847
H	-4.875945	-2.502689	1.984792	H	-6.792650	-3.145027	0.233597
C	-5.401492	-2.058794	-0.136878	C	-4.909752	-2.119769	-0.396327
H	-6.025999	-1.381600	-0.703432	H	-5.058525	-1.731745	-1.408907
H	-5.418074	-3.094493	-0.448533	H	-4.434601	-3.101363	-0.499908
C	-2.948333	-2.473762	0.035286	C	-3.380148	-1.651999	1.506798
H	-3.251692	-3.394654	0.529182	H	-4.106508	-2.276229	2.023917
H	-2.083423	-2.066813	0.546687	H	-3.145249	-0.814807	2.158267
H	-2.688015	-2.678218	-1.000622	H	-2.470712	-2.237715	1.344302
O	-4.829847	0.467112	-0.923027	O	-4.041751	0.236637	-1.502997
C	-2.048478	1.604273	-0.778819	C	-1.932528	1.810070	-0.731223
N	-1.165467	2.572264	-0.121063	N	-1.028609	2.764217	-0.081460
C	-1.756454	3.861763	0.202230	C	-1.594681	4.059553	0.259050
H	-2.722627	3.709970	0.684926	H	-2.520813	3.925870	0.821291
H	-1.127677	4.425377	0.882352	H	-0.913643	4.636889	0.873762
H	-1.919614	4.457407	-0.700209	H	-1.826482	4.635509	-0.640881
C	0.122275	2.229432	0.125243	C	0.264755	2.403713	0.123288
C	1.042302	3.270745	0.733665	C	1.217207	3.426100	0.712588
H	0.749776	3.506487	1.757942	H	0.946653	3.676898	1.739270
H	2.047466	2.862736	0.747559	H	2.211862	2.992879	0.713350

H	1.042558	4.199446	0.163528	H	1.232008	4.350161	0.134786
O	0.542024	1.099813	-0.142013	O	0.657150	1.272403	-0.166951
H	-2.825941	2.161086	-1.298777	H	-2.737944	2.370801	-1.198432
H	-1.468156	1.054526	-1.515910	H	-1.387960	1.296541	-1.519271
